# Progress in Medicine

**Published:** 1895-03-02

**Authors:** 


### Progress in Medicine.

### DIABETES.

**Diet.**—In speaking of the great increase of this complaint of late years, Ralfe points to an important but little noticed change in the national dietary. Cane sugar—once unknown, and then only an occasional condiment—has quite recently become a constant element in English food, while fine flour also and potatos have replaced the mixed meal which was once the staple food of the population.[1] To this change is probably owing much of the existing diabetes. He considers that a strict diet for diabetes must be undertaken with great care, gradually, and while the effect is watched as sugar and other articles are cut off one by one. If this suffices to remove the sugar from the urine entirely, a gradual return after some weeks to ordinary food minus cane sugar may be attempted. In acute cases, however, a strict diet may be needed permanently, especially if the sugar excreted is out of proportion to that consumed. A pure meat diet causes always some risk of poisoning from toxins, and itself contains some carbo-hydrates.

He is most careful to restrict the quantity of food and to introduce variety, while giving plenty of bacon, butter, and cream. Gluten bread contains some 20 per cent. of starch, and should be eaten very moderately, or in most cases four ounces of plain bread may be substituted, preferably toasted. Very little milk, but green vegetables, cocoanut, filberts, lemon juice, and even sometimes tomatos and currants are useful. The alcohol desirable varies much with the state of the patient. He suggests as a daily diet—Meat, 12 oz.; green vegetables, 14; fats, 12; eggs, cheese, &c., 14; gluten flour, 12.

The analysis of Clarke's starchless biscuits, the "Ne Far," brand showed that no starch was present but 49 per cent. of albuminoids.[2] Elvey advocates also a diet graduated according to the results on the urine, with careful watching in the intermittent cases, and the use of alkaline waters, quinine, and cold affusions to the head.[3] Monin believes that permanganate of potash, gr. ½ to gr. 1½, twice daily, with the copious use of hot maté tea for a beverage, will often save the necessity for dieting in gouty glycosuria. The powdered maté should be drunk[4] with the infusion, and acts like opium in reducing the amount of sugar. In these arthritic cases, Barth, too, lays stress[5] on the injuriousness of a strict diet, since the real evil lies in faulty assimilation, not in the over-production, of sugar. Hence he prescribes iodides, salicylates, and alkalies, with a diet including milk, eggs, and a little bread. He divides neuropathic diabetes, again, from the malignant form, where alone he gives strict meat diet and opium. It is remarkable how little use has been made of desiccated cocoanut, which only costs 4½d. a pound, as well as of Iceland moss and the German substitute for gluten, aleuronal, which is also inexpensive. Most palatable and safe dishes can be made from them.[6] Solis Cohen[7] keeps his patients on a diet only restricted enough to diminish or abolish the glycosuria without making the patient uncomfortable, and finds lævulose sugar assimilated and useful. The alkalies, codein, and hydrogen peroxide are drugs on which most reliance can be placed. The value of lævulose has been further tested by Grube and Hale White. The former[8] regards it as assimilable in mild cases; Hale White finds that, when large quantities are given,[9] some appears in the urine, and some of it is certainly assimilated. None of the patients felt worse, and some gained in weight; in none of them was the sugar excretion stimulated beyond the amount given, and in some there was an actual decrease. Haycraft found 50 grammes or more utilised daily in a chronic glycosuria.[10] It is clear, however, that in severe cases it must be used cautiously.

**Therapeutics.**—Apart from dietetic treatment, the drugs most discussed have been guiacol, piperazine, and minute doses of pilocarpine for thirst. Lenné[11] finds jambul bark useless, like the fruit. P. Watson Williams reported a case of much interest, where grafts of a sheep's pancreas were inserted into the subcutaneous tissue, after trial had been previously made in vain of ioderic pancreatic extract and other remedies. Diabetic coma, however, came on,[12] and the patient died. The pancreas was found almost entirely atrophied, which afforded a strong justification for the procedure. Where pancreatic diseases can be diagnosed, transplantation offers the most rational mode of treatment. McNamara suggests the injection per rectum of fresh pancreatic extract,[13] that it may enter the portal vessels direct. He proposes injections larger than would be carried off by the lymphatics, or even hypodermic injections in the same region. Clemens reported a great reduction of the sugar and urine after a week's administration of guiacol in doses of 5 to 10 drops three times a day.[14] Piperazin is recommended by Hildebrandt[15] as lessening the activity of the amylolytic ferment, and in phloridzin diabetes he obtained good results. These do not, however, seem to have been since confirmed in clinical cases. The drug, however, must be given on an empty stomach, with a full dose of an alkali to neutralise any acidity.

**Complications.**—As a symptom of approaching coma the occurrence of casts in the urine is of importance. Kutz found them[16] in 400 cases, confirming the previous observations of Sandmeyer in this point. The presence of albumen is almost exclusively met with in gouty glycosuria, but may be found during coma in any form of the disease. The less serious type of albuminuria is intermittent, and the general health remains good; in the grave type all the symptoms of nephritis follow. The quantities of the sugar and albumen often bear no fixed relation to one another, but the substitution of albumen for sugar is always a grave sign. The kidney lesions may be interstitial sclerosis, or the hyaline degeneration of the epithelium from infiltration with glycogen, which Ehrlich believes to be constantly present in diabetes. Jacobson[17] holds that the important matter in treatment is the general health of the patient. The mild type must be treated as diabetes only with alkalies, baths, &c. In the severe form, neither a milk nor a pure meat diet are admissible on account of the two diseases present. The iodides and phosphate of lime are sometimes serviceable. Indeed, these latter cases present very serious difficulties, but the safest course seems to be to treat the kidney disease by ordinary methods without attempting a milk diet.

The frequent danger of sudden heart failure, especially when renal disease is also present, was generally admitted in a debate at the congress of American physicians and surgeons[18]. Stephen Mackenzie reported that forty-five out of eighty cases under his care died suddenly. Hence the importance of not regarding merely the sugar, but the general health of the patient. Surgical intervention during diabetes must be avoided, if possible, from the effect in the heart, the slowness with which diabetic tissues[19] heal and the danger from the anæsthetic. It may also rouse a quiescent glycosuria. Becker insists on previous strict diet and equally strict asepsis. Reynier thinks that operations should be avoided[20] if the reflexes are absent, or if more than 300 grains of sugar are excreted daily. In gouty glycosuria there is less risk. Asepsis, not antiseptics, must be rigidly maintained in every case or gangrene and other complications will result. An important and comprehensive review of the subject is given by Smith, Bellingham, and Durham,[21] and the effect of injuries to the head and spine, of neoplasms and abscesses in exciting glycosuria is also made clear. These traumatic cases generally lose their glycosuria after a time and the primary wound is not affected by it. A well-marked case of glycosuria following the use of thyroid extract[22] and ceasing with its withdrawal is reported by Dale James. Others appear with an attack of appendicitis and generally pass away after the peritoneal lesion is cured. Four such instances are discussed by Leidy[23] and Solis Cohen,[24] one of which was successfully operated upon. A mild form sometimes occurs in the secondary incubation stage of syphilis,[25] but there is little increase in the urine, and the disorder passes away spontaneously, or under anti-syphilitic treatment. It has, again, been found to follow recurrent nasal obstruction, as in a case of Bayes's,[26] who is inclined to regard these cases as caused by reflex nervous action rather than from the diminution of the oxygen inhaled.

The extraordinary tolerance of diabetics for opium is shown by Broadbent,[27] who administered eight grains of codeia, and, again, six grains of opium daily to a child of four-and-a-half years with no soporific effect, and, indeed, with little result on the excretion of sugar. Another point useful for diagnosis is the frequent occurrence of cramps in the calves in early stages of the disease. Unschuld found it in nearly a third of his patients.[28] Whether it is caused directly by the sugar in the blood, or by microbic toxins, so easily introduced in diabetics, is not known.

[1] Clin. Jour., Oct. 17th. [2] B. M. J., May 5th. [3] Jour. des Prac., Nov. 8th, 1894. [4] Med. Rec., April 8th. [5] Med. Week, Aug. 3rd. [6] Med. Chron., May. [7] Ther. Gaz., Aug. 15th. [8] Med. Chron., Nov. [9] Pract., Oct. [10] Med. Chron., Oct. [11] Ther. Monats., May. [12] B. M. J., Dec. 8th. [13] B. M, J., July 21st. [14] Med. Chron., June. [15] B. M. J., March 31st. [16] Amer. M. and S. Bullet., Oct. [17] Gaz. des Hop., July 25th. [18] Med. Rec., July 28th. [19] Am. J. Med. Sc., Dec. [20] Ther. Gaz. [21] Guy's Hosp., Rep., Vol. 49, [22] B. J., Bermatol, June. [23] B. M. J., Nov. 10th. [24] N. Y. Med., Aug. 11th. [25] B. M. J., Sep. 1st. [26] B. M. J., Oct. 3rd. [27] Lanc., Sep. 15th. [28] B. M. J., Aug. 11th.

